# Significant association between *rs28362491* polymorphism in *NF-κB1* gene and coronary artery disease: a meta-analysis

**DOI:** 10.1186/s12872-020-01568-0

**Published:** 2020-06-08

**Authors:** Yanwei Wang, Bianwen Wu, Muqing Zhang, Huawei Miao, Jiaan Sun

**Affiliations:** 1 Department of Cardiology, Hebei Province Hospital of Traditional Chinese Medicine, Zhongshan East street 389#,, Shijiazhuang, 050011 Changan District China; 2 Department of Cardiology, 980 Hospital of PLA joint Logisitics Support Force, Shijiazhuang, 050000 China

**Keywords:** *NF-κB1*, *Rs28362491*, Coronary artery disease, Polymorphism, Meta-analysis

## Abstract

**Background:**

The association of *rs28362491* polymorphism in *NF-κB1* gene and coronary artery disease (CAD) risk was reported in several studies with inconsistent outcomes. This study aimed to comprehensively collect and synthesize the existing evidence to appraise whether *rs28362491* was correlated to CAD susceptibility.

**Methods:**

Databases of Web of Science, EMBASE, PubMed, Wanfang, and CNKI were retrieved from inception to August 1, 2019 without any restriction on language. The strengths of association between *rs28362491* polymorphism and CAD were presented as odds ratios (ORs) and 95% confidence intervals (CIs).

**Results:**

Thirteen case-control studies with 17 individual cohorts containing 9378 cases and 10,738 controls were incorporated into this meta-analysis. The findings indicated that *rs28362491* polymorphism was significantly correlated to CAD risk in five genetic models: D vs. I, OR = 1.16, 95%CI 1.11–1.21, *P<*0.01; DD vs. II, OR = 1.37, 95%CI 1.25–1.49, *P<*0.01; DI vs. II, OR = 1.11, 95%CI 1.05–1.18, *P*<0.01; DD + DI vs. II, OR = 1.17, 95%CI 1.11–1.24, *P*<0.01; DD vs. DI + II, OR = 1.29, 95%CI 1.15–1.43, *P*<0.01. After stratification by ethnicity and gender, significant association still existed between *rs28362491* and CAD, especially in the dominant model.

**Conclusions:**

The findings suggest that the mutant D allele in *rs28362491* locus may increase the risk of CAD, and carriers of D allele appear to be more susceptible to CAD.

## Background

Coronary artery disease (CAD), which consists of clinical manifestations including stable or unstable angina, myocardial infarction, and sudden coronary death, is a leading cause of disability and death worldwide [1, 2]. The prevalence of CAD is reported to be 4.6–9.2% in the overall population, and 11.3–31.3% among the elderly aged over 65 [3]. Due to its high prevalence and incapacity, CAD has become a major health concern and imposed a heavy burden on society [2, 4].

A range of environmental factors like smoking [5], unhealthy diet and lifestyle [6], lack of physical activity [7], and low socioeconomic status [8], have been reported to stimulate the occurrence and progression of CAD. The large majority of CAD, nevertheless, are multifactorial with both environmental and heritable contributions [9]. Familial aggregation of CAD has been observed for a long time, and overwhelming evidence indicated the genetic predisposition to CAD [10, 11]. Twin studies revealed the heritability estimates for CAD ranged from 41 to 77% [12]. The importance of genetic factors in CAD resulted in extensive identification of considerable candidate genes and numerous single-nucleotide polymorphisms (SNPs) that were related to this condition [13].

Nuclear factor-κB (NF-κB) is a cluster of ubiquitous transcription factors. In the NF-κB family, there are five members including NF-κB1, RelA, c-Rel, RelB, and NF-κB2. As a fundamental transcription regulator, NF-κB participates in most biological processes. The NF-κB signaling pathway also plays a crucial role in heart diseases [14]. NF-κB1 is encoded by *NF-κB1* gene, which is mapped to chromosome 4q24. *Rs28362491* polymorphism is a 4 bp ATTG insertion/deletion variation in -94 bp of *NF-κB1* promoter. This polymorphic site was reported to have great impact on the expression of *NF-κB1* gene, with the deletion of ATTG causing reduced promoter activity.

Xie et al. [15] initially reported the correlation between *rs28362491* polymorphism and risk of CAD in a Chinese cohort, investigators attempted to conduct replicate studies in different populations with inconsistent results. The conflicting findings might ascribe to small sample size and weak statistical power of an individual study. Therefore, we carried out this meta-analysis to obtain more convincing evidence on the association of *rs28362491* polymorphism and CAD susceptibility.

## Methods

### Search strategy of literature

We searched databases of Web of Science, EMBASE, PubMed, Wanfang, and CNKI before August 1, 2019. For PubMed database, the search string was (“Polymorphism, Genetic”[Mesh] OR polymorphism OR Single Nucleotide Polymorphism OR Genetic Polymorphism OR Mutant OR Variant) AND (“NF-kappa B p50 Subunit”[Mesh] OR NFKB1 OR NF-kappa B p50 Subunit OR NF-κB1 OR NF-kappa B p50 OR NF kappa B p50) AND (Acute coronary syndrome OR Coronary Artery Disease OR Coronary Arteriosclerosis OR Coronary Atherosclerosis OR “Coronary Artery Disease”[Mesh]). No language restriction was set during the literature search process. The reference lists of relevant studies were manually examined to obtain more publications.

### Inclusion and exclusion criteria

Identified studies met the following criteria were included: (i) case-control or cohort studies investigating *rs28362491* and CAD; (ii) confirmed diagnosis of CAD for cases; (iii) studies with available data to calculate odds ratios (ORs) and 95% confidence intervals (95%CIs); (iv) studies that conformed to Hardy-Weinberg equilibrium (HWE) [16]. Accordingly, editorial, review, conference abstract, and animal study were excluded.

### Quality assessment

Quality assessment was carried out by two investigators independently (YW and BW) based upon Newcastle-Ottawa Scale (NOS) [17]. The quality of each study was judged by three aspects including selection (4 items), comparability (2 items), and exposure (3 items). Scores of 0–3, 4–6, and 7–9 suggested low-, moderate-, and high-quality, respectively.

### Data extraction

The following data were collected by two investigators independently (YW and BW): (i) first author; (ii) date of publication; (iii) nationality of participants; (iv) ethnicity; (v) gender; (vi) sample size; (vii) genotype distribution; (viii) results of HWE test. Any disagreement was resolved until reaching a consensus.

### Statistical analysis

The association of *rs28362491* polymorphism and CAD risk was presented as ORs and 95%CIs in allelic model (D vs. I), homozygous model (DD vs. II), heterozygous model (DI vs. II), dominant model (DD + DI vs. II), and recessive model (DD vs. DI + II). Q-statistical test and I^2^ test were used to examine the between-study heterogeneity. *P* < 0.1 and I^2^ > 50% indicated the heterogeneity was considerable, and a random-effects model should be applied, otherwise a fixed-effects model could be employed. Subgroup-analyses by ethnicity and gender were performed to test if there were ethnicity-specific and gender-specific effects. The data analyses were accomplished using RevMan 5.3 software.

### Sensitivity analysis and publication bias

Stability of the pooled results was detected through sensitivity analysis by removing each dataset in turn and recalculating the effect sizes. Publication bias was examined by funnel plots.

## Results

### Literature search

**Fig. 1:**
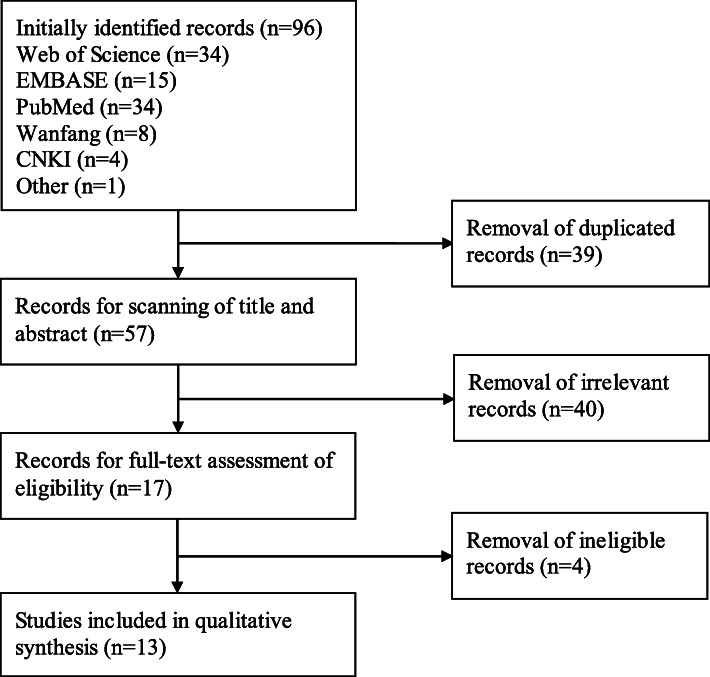
Flow chart of literature search and screen

Ninety-six items were obtained after the search of five databanks and reference lists of relevant studies. Thirty-nine items were deleted because of duplication. Of the remaining 57 items, 40 obviously irrelevant citations were removed after the screen of titles and abstracts. After that, another four ineligible articles were deleted after comprehensive assessment. Finally, 13 studies with 17 individual cohorts were included in the final meta-analysis. The procedures of literature search and selection were displayed in Fig. 1.

### Main characteristics

Main characteristics of the eligible studies were summarized in Table 1. Thirteen studies [15, 18–29] with 17 individual cohorts containing 9378 cases and 10,738 controls were incorporated into this meta-analysis. Of them, 11 studies [19–29] were published in English and the rest two studies [15, 18] were in Chinese. The studies by Yang et al. [26], Stegger et al. [28], and Mishra et al. [29] consisted of two, three, and two individual cohorts, respectively. The included studies were conducted in different countries including China, Iran, Spain, Turkey, Denmark, USA, and India. Of note, the diagnosis of CAD was through coronary angiography by all included study with the exception of Stegger et al. [28], in which CAD was defined as unstable angina pectoris, myocardial infarction, and fatal coronary heart disease. All the studies conformed to HWE. In the light of NOS, all eligible studies obtained 5 to 9 stars, indicating a moderate to high quality (Table 2).

**Table 1:** Main characteristics of included studies

Study	Year	Country	Ethnicity	Gender	Sample size	Case				Control			HWE
						DD	DI	II		DD	DI	II	
Hu XJ	2019	China	Asian	Both	182/307	36	81	65		40	147	120	0.63
Jin SY	2019	China	Asian	Both	778/1112	140	387	251		157	529	426	0.72
Coto E	2019	Spain	Caucasian	Male	609/423	91	291	227		59	201	163	0.81
Seidi A	2018	Iran	Asian	Both	124/100	27	50	47		9	38	53	0.57
Luo JY	2017	China	Asian	Both	1184/1112	215	562	407		157	529	426	0.72
Lai HM	2015	China	Asian	Both	960/1060	178	425	357		131	492	437	0.70
Lai H	2015	China	Asian	Both	1140/1156	218	530	392		154	561	441	0.24
Arslan S	2015	Turkey	Asian	Both	226/201	36	114	76		20	96	85	0.34
Mishra A	2014	India	Asian	Both	510/230	46	166	298		15	68	147	0.07
Yang YN (I)	2014	China	Asian	Both	633/616	126	282	225		103	291	222	0.64
Yang YN (II)	2014	China	Asian	Both	437/356	70	195	172		48	161	147	0.71
Stegger JG (I)	2013	Denmark	Caucasian	Both	1012/1719	171	484	357		255	792	672	0.39
Stegger JG (II)	2013	USA	Caucasian	Male	427/873	57	216	154		140	389	344	0.09
Stegger JG (III)	2013	USA	Caucasian	Female	471/922	75	213	183		148	412	362	0.09
Mishra A (I)	2013	India	Asian	Both	310/230	27	96	187		15	68	147	0.07
Mishra A (II)	2013	India	Asian	Both	290/230	28	99	163		15	68	147	0.07
Xie FY	2008	China	Asian	Both	85/88	18	41	26		22	41	25	0.53

*HWE* Hardy-Weinberg Equilibrium

**Table 2:** Quality assessment of included studies according to the Newcastle-Ottawa Scale

Item/Study	Adequate definition of cases	Representativeness of cases	Selection of control subjects	Definition of control subjects	Control for important factor	Exposure assessment	Same method of ascertainment for all subjects	Non-response rate	Total
Hu XJ, 2019 [18]	1	0	0	1	1	1	1	1	6
Jin SY, 2019 [19]	1	0	0	1	2	1	1	1	7
Coto E, 2019 [20]	1	0	1	1	1	1	1	1	7
Seidi A, 2018 [21]	1	0	0	1	1	1	1	1	6
Luo JY, 2017 [22]	1	0	0	1	2	1	1	1	7
Lai HM, 2015 [23]	1	0	1	1	2	1	1	1	8
Lai H, 2015 [24]	1	0	1	1	2	1	1	1	8
Arslan S, 2015 [25]	1	0	0	1	2	1	1	1	7
Yang YN, 2015 [26]	1	0	1	1	2	1	1	1	8
Mishra A, 2013 [27]	1	0	0	1	2	1	1	1	7
Stegger JG, 2013 [28]	1	1	1	1	2	1	1	1	9
Mishra A, 2013 [27]	1	1	0	1	2	1	1	1	8
Xie FY, 2008 [15]	1	0	0	1	1	1	1	1	6

### Meta-analysis and subgroup-analysis

**Fig. 2:**
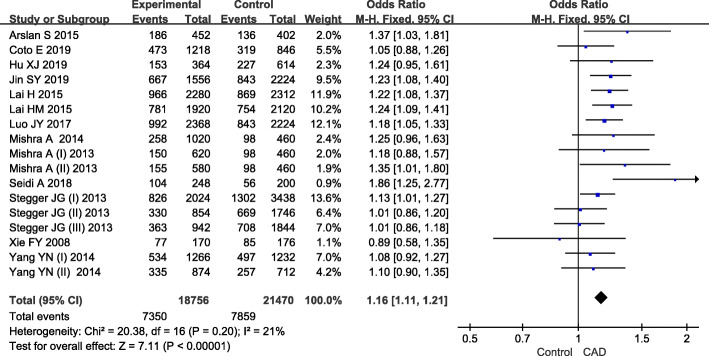
Forest plot of association between *rs28362491* and coronary artery disease in allelic model

Results of overall and subgroup analyses were summarized in Table 3. For the overall populations, between-study heterogeneity was observed in recessive model, and the random-effects model was employed. For the rest contrasts, little heterogeneity was found and the fixed-effects model was used. The pooled data indicated that *rs28362491* was significantly associated with increased risk of CAD under all five genetic models: D vs. I, OR = 1.16, 95%CI 1.11–1.21, *P*<0.01 (Fig. 2); DD vs. II, OR = 1.37, 95%CI 1.25–1.49, *P*<0.01; DI vs. II, OR = 1.11, 95%CI 1.05–1.18, *P*<0.01; DD + DI vs. II, OR = 1.17, 95%CI 1.11–1.24, *P*<0.01; DD vs. DI + II, OR = 1.29, 95%CI 1.15–1.43, *P*<0.01.

**Table 3:** Association between *rs28362491* polymorphism and coronary artery disease

Genetic model	No. of cohorts	Association			Effect model	Heterogeneity	
		OR	95%CI	P-value		I^2^ (%)	P-value
Overall							
D vs. I	17	1.16	1.11–1.21	<0.01	F	21	0.20
DD vs. II	17	1.37	1.25–1.49	<0.01	F	32	0.10
DI vs. II	17	1.11	1.05–1.18	<0.01	F	0	0.95
DD + DI vs. II	17	1.17	1.11–1.24	<0.01	F	0	0.84
DD vs. DI + II	17	1.29	1.15–1.43	<0.01	R	38	0.06
Asian							
D vs. I	13	1.21	1.15–1.27	<0.01	F	0	0.52
DD vs. II	13	1.50	1.35–1.67	<0.01	F	0	0.58
DI vs. II	13	1.11	1.03–1.20	<0.01	F	0	0.90
DD + DI vs. II	13	1.20	1.12–1.28	<0.01	F	0	0.79
DD vs. DI + II	13	1.43	1.30–1.57	<0.01	F	0	0.67
Caucasian							
D vs. I	4	1.07	0.99–1.15	0.08	F	0	0.58
DD vs. II	4	1.11	0.95–1.29	0.20	F	0	0.44
DI vs. II	4	1.12	1.00–1.25	0.05	F	0	0.67
DD + DI vs. II	4	1.12	1.00–1.24	0.04	F	0	0.72
DD vs. DI + II	4	1.04	0.90–1.20	0.59	F	15	0.31
Female							
D vs. I	8	1.14	1.06–1.24	<0.01	F	14	0.32
DD vs. II	8	1.37	1.16–1.61	<0.01	F	41	0.11
DI vs. II	8	1.06	0.94–1.19	0.36	F	0	0.82
DD + DI vs. II	8	1.13	1.01–1.26	0.04	F	0	0.83
DD vs. DI + II	8	1.37	1.08–1.73	<0.01	R	53	0.04
Male							
D vs. I	9	1.16	1.05–1.27	<0.01	R	55	0.02
DD vs. II	9	1.30	1.07–1.60	0.01	R	59	0.01
DI vs. II	9	1.18	1.04–1.34	<0.01	R	43	0.08
DD + DI vs. II	9	1.21	1.07–1.37	<0.01	R	46	0.06
DD vs. DI + II	9	1.19	0.99–1.42	0.06	R	57	0.02

*OR* Odds ratio, *CI* Confidence interval, *F* Fixed-effects model, *R* Random-effects model

Subgroup analysis by ethnicity revealed that *rs28362491* was significantly associated with an increased CAD risk in Asians under five genetic models: D vs. I, OR = 1.21, 95%CI 1.15–1.27, *P*<0.01; DD vs. II, OR = 1.50, 95%CI 1.35–1.67, *P*<0.01; DI vs. II, OR = 1.11, 95%CI 1.03–1.20, *P*<0.01; DD + DI vs. II, OR = 1.20, 95%CI 1.12–1.28, *P*<0.01; DD vs. DI + II, OR = 1.43, 95%CI 1.30–1.57, *P*<0.01. While, for the Caucasian population, only marginally positive associations were witnessed in heterozygous model (DI vs. II, OR = 1.12, 95%CI 1.00–1.25, *P* = 0.05) and dominant model (DD + DI vs. II, OR = 1.12, 95%CI 1.00–1.24, *P* = 0.04), and no association was observed under other contrasts.

Further subgroup analysis by gender demonstrated that *rs28362491* was significantly associated with an increased risk of CAD in females under allelic model (D vs. I, OR = 1.14, 95%CI 1.06–1.24, *P*<0.01), homozygous model (DD vs. II, OR = 1.37, 95%CI 1.16–1.61, *P*<0.01), dominant model (DD + DI vs. II, OR = 1.13, 95%CI 1.01–1.26, *P* = 0.04), and recessive model (DD vs. DI + II, OR = 1.37, 95%CI 1.08–1.73, *P*<0.01). When it came to males, significant associations were found under allelic model (D vs. I, OR = 1.16, 95%CI 1.05–1.27, *P*<0.01), homozygous model (DD vs. II, OR = 1.30, 95%CI 1.07–1.60, *P* = 0.01), heterozygous model (DI vs. II, OR = 1.18, 95%CI 1.04–1.34, *P*<0.01), and dominant model (DD + DI vs. II, OR = 1.21, 95%CI 1.07–1.37, *P*<0.01).

### Sensitivity analysis and publication bias

**Fig. 3:**
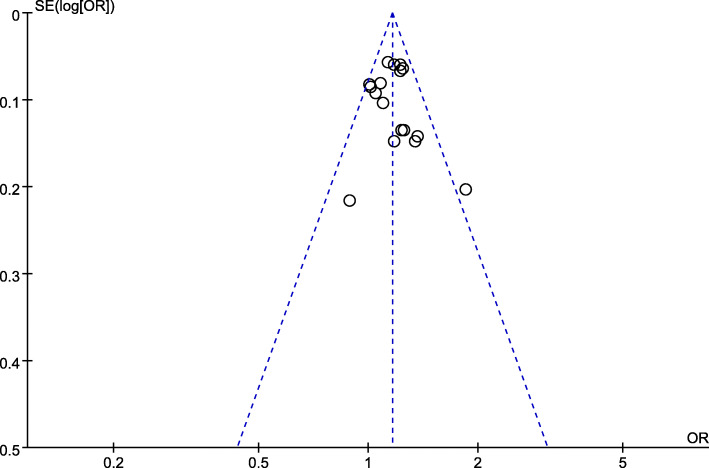
Funnel plot of association between *rs28362491* and coronary artery disease

After removal of each study, the re-calculated effect sizes did not reverse, which confirmed the stability and reliability of our findings. Visual inspection of funnel plots did not identify obvious asymmetry, indicating the outcomes were unlikely to have severe publication bias (Fig. 3).

## Discussion

CAD causes nearly one-third of all deaths in individuals aged over 35 [30]. However, the etiology of CAD remains far from clear. Knowledge of the etiology behind CAD can lead to early preventative modifications and improved therapeutics of this disease. Similar to most diseases, CAD is also a multifactorial disorder. In the past decade, genetic factors were recognized to make substantial contribution to CAD vulnerability and received much more attentions. Genome-wide association studies and case-control studies have recognized considerable genes and polymorphic loci that are associated with CAD risk [13, 31]. *Rs28362491* polymorphism in *NF-κB1* gene is one of the most widely studied locus.

Studies on the association of *rs28362491* polymorphism and CAD predisposition have been carried out in different regions. However, it is quite regrettable that those studies reported inconsistent findings and more convincing evidence remains to be determined. By merging the data from previous studies, this meta-analysis indicated that *rs28362491* was significantly associated with CAD. The findings of subgroup-analyses witnessed that *rs28362491* was significantly associated with CAD in dominant comparison, regardless of ethnicity and gender. Of note, in the Caucasian population, the association was of borderline significance, which might be attributable to limited sample size of participants. In addition, the trends of associations in different genders were not always consistent, which might be caused by gender differences of genotype distributions.

NF-κB is a pleiotropic transcription regulator implicating in diverse biological process including cell proliferation, differentiation, adhesion, and apoptosis, thus exerting crucial effects on multiple pathological states like inflammatory response, which plays a vital role in the development of CAD [32]. Despite different dimeric forms of NF-κB have been reported, NF-κB1 and RelA are the most extensively studied isoforms [33]. *NF-κB1* gene encodes a non-DNA-binding cytoplasmic protein (p105) and a DNA-binding protein (p50) that bind to N-terminus of p105 [34]. Campbell et al. [35] reported that p50 homodimers could impede p65 dimers from combining to promoters and activating genes involved in inflammatory response. The p50 homodimers also promoted the transcription of pro-inflammatory cytokines and inhibited the transcription of anti-inflammatory cytokines.

*Rs28362491* polymorphism is located between two putative key promoter regulatory elements of *NF-κB1* gene. This polymorphic site consists of three genotypes, namely mutant homozygous DD (deletion/deletion), heterozygous DI (deletion/insertion), and wild homozygous II (insertion/insertion) [36]. Park et al. found that individuals with DD homozygous genotype have lower activated NF-κB [37]. Additionally, compared with II homozygous endothelial cells, DD homozygous cells appeared to have a lower increase in eNOS protein level under unidirectional laminar shear stress [37]. Fontaine-Bisson et al. [38] observed that D allele of *rs28362491* was associated with higher level of C-reactive protein, which was an established risk indicator for cardiovascular disease. The in vitro study revealed that the D allele in *rs28362491* polymorphism might result in reduced *NF-κB1* message, thus decreasing the expression of NF-κB1 [39]. The NF-κB1 appears to be specifically involved in anti-inflammatory effects, and decreased NF-κB1 can promote the transcription of pro-inflammatory cytokines like TNF-α and IL-12 [40], which are contributors of CAD [41, 42].

The findings of this study could contribute to a broader recognition of the genetic architecture of CAD. In the near future, with the enhanced understanding of genetics, evidence from the genetic studies may have the capacity to improve healthcare of an individual with CAD by taking preventive measures, offering accurate diagnosis, as well as providing personalized treatment. Additionally, appropriate screening programs can be carried out for an individual with a first-degree relative has a history of CAD. Genetic studies may serve to recognize genes as potential therapeutic targets.

It was noted that Chen et al. [43] had published a similar meta-analysis in 2014. Nevertheless, the current study has notable advantages. First, several newly published papers [18–22, 24, 25] were included in our study, and the number of included studies and participants were greatly enlarged. Second, a more comprehensive search strategy was employed to obtain relevant studies at a maximum. Third, we adopt a more strict inclusion and exclusion criteria, and excluded Lopez-Mejias et al.’s study [44], of which cardiovascular disease was considered to be equal to CAD by Chen et al. [43]. Fourth, sensitive analysis was conducted to check the stability of the outcomes. Consequently, these advantages strongly guaranteed a more accurate and reliable conclusion.

Inevitably, this study had several drawbacks. First, the effect sizes were based on unadjusted evaluation for lack of sufficient data, and failure to perform further adjusted analyses might influence the reliability of those findings. Second, like most disease, CAD is a multi-factorial disorder that is associated with genetic and environmental factors. Nevertheless, owing to lack of detailed data in primary studies, we were unable to evaluate the effects of multi-factor interactions. Third, the included populations were from Asian and Caucasian populations, ethnicity bias might exist in our analysis. Thus, the findings might not be applicable to other populations. Last, only three English databases and two Chinese databases were retrieved for potentially relevant studies, although no obvious dissymmetry in any funnel plot was observed, publication biases might exist which could influence the results.

## Conclusion

The current study indicates that the mutant D allele in *rs28362491* locus may increase the risk of CAD, and carriers of D allele appear to be more susceptible to CAD. Due to limitations of this study, the association between *rs28362491* polymorphism and CAD could not be entirely concluded. Further well-designed studies among different ethnicities are still warranted to confirm our findings.

## Data Availability

Available upon request to the corresponding author Jiaan Sun, jiaansun@126.com

## Abbreviations

CAD: Coronary artery disease
OR: Odds ratio
CI: Confidence interval
HWE: Hardy-Weinberg equilibrium
NOS: Newcastle-Ottawa scale

## References

[1] Benjamin EJ, Virani SS, Callaway CW, Chamberlain AM, Chang AR, Cheng S (2018). Heart disease and stroke Statistics-2018 update: a report from the American Heart Association. Circulation..

[2] Leong DP, Joseph PG, McKee M, Anand SS, Teo KK, Schwalm JD (2017). Reducing the global burden of cardiovascular disease, part 2: prevention and treatment of cardiovascular disease. Circ Res.

[3] Zhu KF, Wang YM, Zhu JZ, Zhou QY, Wang NF (2016). National prevalence of coronary heart disease and its relationship with human development index: a systematic review. Eur J Prev Cardiol.

[4] Gaziano TA, Bitton A, Anand S, Abrahams-Gessel S, Murphy A (2010). Growing epidemic of coronary heart disease in low- and middle-income countries. Curr Probl Cardiol.

[5] Zhu J, Nelson K, Toth J, Muscat JE (2019). Nicotine dependence as an independent risk factor for atherosclerosis in the National Lung Screening Trial. BMC Public Health.

[6] Stampfer MJ, Hu FB, Manson JE, Rimm EB, Willett WC (2000). Primary prevention of coronary heart disease in women through diet and lifestyle. N Engl J Med.

[7] Huxley RR, Misialek JR, Agarwal SK, Loehr LR, Soliman EZ, Chen LY (2014). Physical activity, obesity, weight change, and risk of atrial fibrillation: the atherosclerosis risk in communities study. Circ Arrhythm Electrophysiol.

[8] Kollia N, Panagiotakos DB, Georgousopoulou E, Chrysohoou C, Tousoulis D, Stefanadis C (2016). Exploring the association between low socioeconomic status and cardiovascular disease risk in healthy Greeks, in the years of financial crisis (2002-2012): the ATTICA study. Int J Cardiol.

[9] Said MA, Verweij N, van der Harst P (2018). Associations of combined genetic and lifestyle risks with incident cardiovascular disease and diabetes in the UK biobank study. JAMA Cardiol.

[10] Mayer B, Erdmann J, Schunkert H (2007). Genetics and heritability of coronary artery disease and myocardial infarction. Clin Res Cardiol.

[11] Fischer M, Mayer B, Baessler A, Riegger G, Erdmann J, Hengstenberg C (2007). Familial aggregation of left main coronary artery disease and future risk of coronary events in asymptomatic siblings of affected patients. Eur Heart J.

[12] Evans A, Van Baal GC, McCarron P, DeLange M, Soerensen TI, De Geus EJ (2003). The genetics of coronary heart disease: the contribution of twin studies. Twin Res.

[13] O'Donnell CJ, Nabel EG (2011). Genomics of cardiovascular disease. N Engl J Med.

[14] Dhingra R, Shaw JA, Aviv Y, Kirshenbaum LA (2010). Dichotomous actions of NF-kappaB signaling pathways in heart. J Cardiovasc Transl Res.

[15] Xie YF, Chen Z, Ma GS, Wang JH, Zhang XL (2008). The chronic inflammation and NF-KB1 gene polymorphism in patients with chronic coronary heart disease. Journal of Clinical Emergency Call.

[16] Ryckman K, Williams SM. Calculation and use of the hardy-Weinberg model in association studies. Curr Protoc Hum Genet. 2008;Chapter 1:Unit 1.18. 10.1002/0471142905.hg0118s57. 10.1002/0471142905.hg0118s57 18428419

[17] Wells GA, Shea B, O’Connell D, Peterson J, Welch V, Losos M (2018). The Newcastle-Ottawa scale (NOS) for assessing the quality of nonrandomised studies in metaanalyses.

[18] Hu XJ (2019). NFKB1 gene polymorphism and early scanning of coronary artery disease. Health Care Today.

[19] Jin SY, Luo JY, Li XM, Liu F, Ma YT, Gao XM (2019). NFKB1 gene rs28362491 polymorphism is associated with the susceptibility of acute coronary syndrome. Biosci Rep.

[20] Coto E, Reguero JR, Avanzas P, Pascual I, Martin M, Hevia S (2019). Gene variants in the NF-KB pathway (NFKB1, NFKBIA, NFKBIZ) and risk for early-onset coronary artery disease. Immunol Lett.

[21] Seidi A, Mirzaahmadi S, Mahmoodi K, Soleiman-Soltanpour M (2018). The association between NFKB1 -94ATTG ins/del and NFKB1A 826C/T genetic variations and coronary artery disease risk. Mol Biol Res Commun.

[22] Luo JY, Li XM, Zhou Y, Zhao Q, Chen BD, Liu F (2017). Mutant DD genotype of NFKB1 gene is associated with the susceptibility and severity of coronary artery disease. J Mol Cell Cardiol.

[23] Lai HM, Li XM, Yang YN, Ma YT, Xu R, Pan S (2015). Genetic variation in NFKB1 and NFKBIA and susceptibility to coronary artery disease in a Chinese Uygur population. PLoS One.

[24] Lai H, Chen Q, Li X, Ma Y, Xu R, Zhai H (2015). Association between genetic polymorphism in NFKB1 and NFKBIA and coronary artery disease in a Chinese Han population. Int J Clin Exp Med.

[25] Arslan S, Korkmaz O, Ozbilum N, Berkan O (2015). Association between NF-kappaBI and NF-kappaBIA polymorphisms and coronary artery disease. Biomed Rep.

[26] Yang YN, Zhang JY, Ma YT, Xie X, Li XM, Liu F (2014). 94 ATTG insertion/deletion polymorphism of the NFKB1 gene is associated with coronary artery disease in Han and Uygur women in China. Genet Test Mol Biomarkers.

[27] Mishra A, Srivastava A, Mittal T, Garg N, Mittal B (2014). Genetic predisposition to left ventricular dysfunction: a multigenic and multi-analytical approach. Gene..

[28] Stegger JG, Schmidt EB, Berentzen TL, Tjonneland A, Vogel U, Rimm E (2013). Interaction between obesity and the NFKB1 - 94ins/delATTG promoter polymorphism in relation to incident acute coronary syndrome: a follow up study in three independent cohorts. PLoS One.

[29] Mishra A, Srivastava A, Mittal T, Garg N, Mittal B (2013). Role of inflammatory gene polymorphisms in left ventricular dysfunction (LVD) susceptibility in coronary artery disease (CAD) patients. Cytokine..

[30] Sanchis-Gomar F, Perez-Quilis C, Leischik R, Lucia A (2016). Epidemiology of coronary heart disease and acute coronary syndrome. Ann Transl Med.

[31] Abraham G, Havulinna AS, Bhalala OG, Byars SG, De Livera AM, Yetukuri L (2016). Genomic prediction of coronary heart disease. Eur Heart J.

[32] Kaptoge S, Seshasai SR, Gao P, Freitag DF, Butterworth AS, Borglykke A (2014). Inflammatory cytokines and risk of coronary heart disease: new prospective study and updated meta-analysis. Eur Heart J.

[33] Blank V, Kourilsky P, Israel A (1992). NF-kappa B and related proteins: Rel/dorsal homologies meet ankyrin-like repeats. Trends Biochem Sci.

[34] Heron E, Deloukas P, van Loon AP (1995). The complete exon-intron structure of the 156-kb human gene NFKB1, which encodes the p105 and p50 proteins of transcription factors NF-kappa B and I kappa B-gamma: implications for NF-kappa B-mediated signal transduction. Genomics..

[35] Low JT, Hughes P, Lin A, Siebenlist U, Jain R, Yaprianto K (2016). Impact of loss of NF-kappaB1, NF-kappaB2 or c-REL on SLE-like autoimmune disease and lymphadenopathy in Fas (lpr/lpr) mutant mice. Immunol Cell Biol.

[36] Ota N, Nakajima T, Shirai Y, Emi M (1999). Isolation and radiation hybrid mapping of a highly polymorphic CA repeat sequence at the human nuclear factor kappa-beta subunit 1 (NFKB1) locus. J Hum Genet.

[37] Park J-Y, Farrance IKG, Fenty NM, Hagberg JM, Roth SM, Mosser DM (2007). NFKB1 promoter variation implicates shear-induced NOS3 gene expression and endothelial function in prehypertensives and stage I hypertensives. Am J Physiol Heart Circ Physiol.

[38] Fontaine-Bisson B, Wolever TM, Connelly PW, Corey PN, El-Sohemy A (2009). NF-kappaB -94Ins/Del ATTG polymorphism modifies the association between dietary polyunsaturated fatty acids and HDL-cholesterol in two distinct populations. Atherosclerosis..

[39] Karban AS, Okazaki T, Panhuysen CI, Gallegos T, Potter JJ, Bailey-Wilson JE (2004). Functional annotation of a novel NFKB1 promoter polymorphism that increases risk for ulcerative colitis. Hum Mol Genet.

[40] Pereira SG, Oakley F (2008). Nuclear factor-kappaB1: regulation and function. Int J Biochem Cell Biol.

[41] Christodoulidis G, Vittorio TJ, Fudim M, Lerakis S, Kosmas CE (2014). Inflammation in coronary artery disease. Cardiol Rev.

[42] Ye J, Wang Y, Wang Z, Liu L, Yang Z, Wang M (2020). Roles and mechanisms of interleukin-12 family members in cardiovascular diseases: opportunities and challenges. Front Pharmacol.

[43] Chen QJ, Lai HM, Zhao L, Ma YT, Li XM, Zhai H (2016). Association between the NFKB1-94ins/del ATTG polymorphism (rs28362491) and coronary artery disease: a systematic review and meta-analysis. Genet Test Mol Biomarkers.

[44] Lopez-Mejias R, Garcia-Bermudez M, Gonzalez-Juanatey C, Castaneda S, Miranda-Filloy JA, Gomez-Vaquero C (2012). NFKB1-94ATTG ins/del polymorphism (rs28362491) is associated with cardiovascular disease in patients with rheumatoid arthritis. Atherosclerosis..

